# Antibiotic Resistance Profiles and Genetic Determinants of *Listeria innocua* Isolated from Food Sources in Poland

**DOI:** 10.3390/genes16121455

**Published:** 2025-12-05

**Authors:** Anna Zawiasa, Agnieszka Olejnik-Schmidt

**Affiliations:** Department of Food Biotechnology and Microbiology, Poznan University of Life Sciences, Wojska Polskiego 48, 60-627 Poznan, Poland; anna.zawiasa@up.poznan.pl

**Keywords:** *Listeria innocua*, antimicrobial resistance, antibiotic, resistance genes

## Abstract

**Background:** Antimicrobial resistance (AMR) is a growing public health concern affecting both medicine and food safety. While *Listeria monocytogenes* is the primary pathogen of concern, *Listeria innocua*—commonly found in food and food-processing environments—may serve as a reservoir for resistance genes and a useful indicator of species for surveillance. This study aimed to assess the phenotypic antibiotic susceptibility and detect resistance-associated genes in *L. innocua* isolates from meat products and processing environments in Poland. **Methods:** A total of 51 *L. innocua* isolates were analyzed, originating from raw and processed meat products as well as meat-processing environments. Antimicrobial susceptibility was determined using the disc diffusion method against 18 antibiotics representing multiple classes. Phenotypic resistance was interpreted following CLSI guidelines (CLSI, 2020). Isolates exhibiting resistance or intermediate resistance were further screened for resistance-associated genes using PCR. **Results:** All isolates were fully susceptible to ampicillin, benzylpenicillin, chloramphenicol, gentamicin, rifampin, trimethoprim-sulfamethoxazole, and vancomycin. High susceptibility was observed for ciprofloxacin, erythromycin, meropenem, trimethoprim, and nitrofurantoin, with only sporadic intermediate responses. Moderate resistance levels were noted for streptomycin (10%) and tetracycline (12%). The lowest susceptibility was recorded for clindamycin and linezolid, with most isolates exhibiting intermediate or resistant phenotypes. Universal resistance to cefotaxime and oxacillin was found. Eighteen distinct resistance patterns were identified. PCR confirmed the presence of several resistance-associated genes, including *mecA*, *lnuA*, *lnuB*, *cfr*, *optrA*, and *poxtA*, consistent with observed phenotypes. **Conclusions:** This study provides the first detailed characterization of AMR in *L. innocua* from Polish meat and processing environments. The findings highlight its heterogeneous resistance profiles and potential role as a reservoir of clinically relevant resistance genes. Incorporating *L. innocua* into surveillance programs may strengthen early detection of emerging resistance and enhance food safety monitoring.

## 1. Introduction

Antimicrobial resistance (AMR) is one of the most critical public health threats of the 21st century [1,2], now ranked among the leading global causes of death [3]. AMR compromises infection treatment but is also a major contributor to significant economic losses, increasing healthcare costs, and reducing productivity. Over time, bacteria have developed resistance to nearly all newly approved antibiotics, posing a serious challenge to modern medicine [1]. Key drivers of resistance include overprescription, self-medication, easy availability of antibiotics, public misconceptions, and widespread use in agriculture [1,4]. The antibiotic sensitivity profile can vary depending on antimicrobial usage patterns across geographic regions [4,5]. Although the use of antibiotics for growth promotion in agriculture has been banned in the EU since 2006 [5], it remains common in other regions, including the U.S., raising concerns about resistance transmission from farm to fork [1]. Global antibiotic use in livestock is projected to increase by 67% between 2010 and 2030, driven by rising demand for animal protein and intensified production systems [6]. In recent years, the environment, particularly soil, which harbors resistance genes due to the natural production of antibiotics by soil-dwelling microbes, has been recognized as a significant contributor to the spread of resistance [7]. Antibiotic resistance commonly arises as a natural process when microorganisms are exposed to antimicrobials exposure. Under selective pressure, susceptible bacteria are eliminated, while resistant isolates survive and proliferate [5,8]. In most bacterial species, resistance arises either through mutations in intrinsic chromosomal genes or the acquisition of new resistance determinants through horizontal gene transfer (HGT) mechanisms such as conjugative plasmids or transposons [9,10]. This genetic exchange facilitates and accelerates the spread of antimicrobial resistance across microbial communities.

Given the growing threat of AMR, coordinated global action is essential to prevent a healthcare crisis [11]. The One Health approach, integrating human, animal, and environmental health, offers a comprehensive framework for addressing antimicrobial resistance and effectively identifies patterns in its emergence and persistence among foodborne pathogens [6,11]. Key solutions to mitigate AMR include raising global awareness and strengthening surveillance of drug resistance and antibiotic efficacy, as well as reducing unnecessary antimicrobial use, especially by restricting access to over-the-counter antibiotics. Innovation also plays a crucial role. Promoting rapid diagnostic tools can help differentiate bacteria from viral infections, could reduce inappropriate antibiotic use. Efforts should focus on improving existing treatments and developing new antibiotics and alternative therapies. Alarmingly, only two new antibiotic classes have been introduced in the past 30 years. Expanding vaccine coverage for bacterial disease coverage can further reduce infection rates and, consequently, the demand for antibiotics [1,11]. Equally important is reducing the non-therapeutic use of antibiotics in animal agriculture, as there is a clear link between antibiotic use on farms and the emergence of resistance in human populations.

Antimicrobial resistance is a critical issue for both medicine and food safety, with resistant strains able to survive across the food production chain. Foodborne diseases remain a major public health concern, as microbial contamination can occur at any stage of the food production chain [12]. *Listeria* spp. is a Gram-positive bacteria widely distributed in the natural environment [8,9,13,14]. Currently, 27 species are recognized within the genus *Listeria* [15], with six considered most relevant: *L. monocytogenes*, *L. ivanovii*, *L. innocua*, *L. grayii*, *L. seeligeri*, and *L. welshimeri* [5,9,16]. While *L. monocytogenes* is the primary cause of listeriosis [2], cases involving *L. ivanovii* and atypical *L. innocua* have also been reported [17,18]. Listeriosis is one of the most severe foodborne diseases affecting humans, typically resulting from the consumption of contaminated food [2,9,19,20]. It is frequently linked to various meat products [21]. Between 1987 and 2018, 2087 global cases of listeriosis were attributed to meat items [9,13]. The EFSA report also noted 35 foodborne outbreaks in 2022, primarily linked to pork, pork-derived products, and fish [22]. Despite improved control strategies, especially in meat products, the incidence of listeriosis has remained stable over the past decade, underscoring the need for continued microbiological monitoring [13,23]. Listeriosis has a significantly higher fatality rate, exceeding 20–30% [13] compared to other foodborne diseases such as campylobacteriosis (0.03%) and salmonellosis (0.22%) [14,24]. Despite its low incidence in the general population, its high mortality rate makes it a significant public health concern [4,13,23].

While *L. monocytogenes* is the primary pathogen of concern, *L. innocua* plays an important role in food safety monitoring and may contribute to the dissemination of antimicrobial resistance. *L. innocua* is a bacterium commonly found in food processing facilities [25], mainly in meat and meat products [21]. Among *Listeria* species, *L. innocua* is the most frequently detected in food products and processing environments [14,21,26], commonly coexisting with *L. monocytogenes* [27,28]. Although generally considered non-pathogenic, *L. innocua* shares an evolutionary lineage with *L. monocytogenes*, having lost key virulence genes [29]. Rare, atypical strains of *L. innocua* carrying pathogenicity island elements have been linked to illness in humans [17,18] and animals [28]. Despite its prevalence, routine diagnostics primarily focus on *L. monocytogenes*, often leading to the omission of *L. innocua* during microbiological testing. Moreover, its ability to dominate selective enrichment media may mask the presence of pathogenic strains, potentially leading to false-negative results or an underestimation of contamination levels [21]. Given its high occurrence, *L. innocua* is increasingly recognized as a useful indicator organism in food safety surveillance [4,28]. Due to its high prevalence and close phenotypic and genotypic similarity to *L. monocytogenes*, *L. innocua* may serve as a reservoir of antimicrobial resistance genes that can be transferred to other *Listeria* species through horizontal gene transfer. In addition, its widespread occurrence makes it a useful indicator of selective pressures present in food production environments. Despite its relevance, data on the antimicrobial susceptibility of *L. innocua* remain scarce. Therefore, monitoring resistance patterns in *L. innocua* is essential for understanding potential risks and improving food safety control strategies.

Due to the high mortality associated with invasive listeriosis, antibiotic treatment is essential to improve clinical outcomes [5,9,24,30]. The standard treatment for listeriosis typically involves the use of ampicillin or penicillin in combination with aminoglycosides such as gentamicin [25,31]. As an alternative, trimethoprim with sulfamethoxazole alone or in combination is recommended as a second-line therapy [11,13,19,32]. In specific cases, it can be replaced by erythromycin, vancomycin, meropenem, rifampicin, and linezolid [32,33,34]. Although *Listeria* spp. is generally susceptible to many antibiotics, excluding cephalosporins, sulfonamides and quinolones, resistance to commonly used agents has been increasingly reported [35]. Instances of *Listeria* spp. resistance to one or more antimicrobial agents have been identified in both human samples and in food processing facilities [9,13]. Treatment failures linked to resistance are largely resulting from mutations and to HGT processes through which *Listeria* acquires resistance genes from other organisms, mainly *Enterococcus* spp. and *Staphylococcus* spp. [34]. Understanding the antibiotic susceptibility of *Listeria* spp. is crucial for effective surveillance and food safety. So far, most studies have focused on the antibiotic resistance of *L. monocytogenes*; however, resistance monitoring should extend beyond this species, as *L. innocua* can itself pose a risk of causing listeriosis and may also act as a reservoir for resistance genes that can be transferred across species [27,36]. Moreover, due to its widespread presence in food and food-processing environments, *L. innocua* can serve as a valuable indicator organism for monitoring antimicrobial resistance trends across the food chain and may provide early warning signals of emerging resistance within *Listeria* populations. Previous studies reported antimicrobial resistance in 19.5% of *L. innocua* isolates compared to only 0.6% of *L. monocytogenes* [37]. Similarly, higher overall resistance rates were observed in *L. innocua* (36.1%) than in *L. monocytogenes* (34.1%) [33], confirming the tendency of *L. innocua* to exhibit greater antimicrobial resistance levels. These findings indicate potential interspecies of differences in antimicrobial susceptibility among *Listeria* species. Despite the significance of understanding the prevalence of antibiotic-resistant *L. innocua*, there remains a notable lack of published research on the antimicrobial susceptibility of strains isolated from food and food-processing environments in Poland. To date, no large-scale studies have been conducted in this area. Consequently, systematic surveillance of resistance - however infrequent - remains essential.

## 2. Aim of Study

The objective of this study was to investigate the antibiotic susceptibility profiles of *L. innocua* isolates obtained from meat products and meat processing environments in Poland, to 18 selected antibiotics, as well as to characterize genetic resistance determinants.

## 3. Materials and Methods

### 3.1. Bacterial Isolates and Genetic Material

Isolates were obtained from the microbiological collection of the Department of Biotechnology and Food Microbiology at the Poznan University of Life Sciences, previously isolated during routine microbiological analyses of food. The isolates were collected between October 2020 and November 2021. A total of 51 *L. innocua* isolates were analyzed in this study, sourced from raw (poultry, pork, beef) and processed meat products (sausage, ham, bacon) (n = 26), other meat sources (n = 6), as well as from the meat-processing environment (n = 19) in Poland. Species identification of the isolates as *L. innocua* was previously confirmed through two genetic techniques: PCR-RFLP [38] and multiplex PCR following the protocol [39]. Methodological details have been previously published [40]. Isolates preserved in Brain Heart Infusion (BHI) glycerol stocks at −80 °C were used for antibiotic susceptibility testing via the disk diffusion method. Genomic DNA for gene detection was extracted using the Genomic Mini Kit (A&A Biotechnology, Gdansk, Poland) following the manufacturer’s protocol and subsequently stored at −20 °C.

### 3.2. Antimicrobial Susceptibility Testing—Disk Diffusion Method

Antimicrobial susceptibility was determined using the diffusion method according to the standard procedure described by the Clinical and Laboratory Standards Institute (CLSI, 2020) [41] and previously applied in studies on *L. monocytogenes* [42]. The *L. innocua* isolates preserved in glycerol stocks were cultured on BHI agar plates Oxoid, Warsaw, Poland) and incubated at 35 °C for 24 h. Single colonies from the BHI agar were used to inoculate 5 mL of liquid BHI medium (Oxoid, Warsaw, Poland) and incubated at 35 °C for 24 h. Post-incubation, the cultures were centrifuged, and supernatants discarded. The bacterial pellets were resuspended in sterile distilled water to prepare bacterial suspensions standardized to 0.5 ± 0.05 McFarland. The suspension was used to inoculate Mueller-Hinton agar plates (Oxoid, Warsaw, Poland) by three-directional streaking. Antibiotic susceptibility was assessed using the disk diffusion method. Antibiotic-impregnated disks (Oxoid, Warsaw, Poland) were aseptically placed on the inoculated agar (up to five disks per plate) and incubated at 35 °C for 24 h. The antibiotics evaluated in this study included: β-lactams: ampicillin (AMP, 10 µg), benzylpenicillin (P, 10 IU), cefotaxime (CE, 30 µg), oxacillin (OX, 1 µg), meropenem (MEM, 10 µg); Aminoglycosides: gentamicin (CN, 10 µg), streptomycin (S, 10 µg); Macrolides and lincosamides: erythromycin (E, 15 µg), clindamycin (DA, 2 µg); Tetracyclines: tetracycline (TE, 30 µg); Fluoroquinolones: ciprofloxacin (CIP, 5 µg); Phenicols: chloramphenicol (C, 30 µg); Oxazolidinones: linezolid (LNZ, 10 µg); Rifamycins: rifampin (RA, 5 µg); Nitrofurans: nitrofurantoin (F, 300 µg); Folate inhibitors: trimethoprim (TMP, 5 µg) and trimethoprim/sulfamethoxazole (SXT, 1.25/23.75 µg) and Glycopeptides: vancomycin (VA, 30 µg). Zones of inhibition were measured in millimeters, with each zone measured three times and the average calculated. Testing was performed in two independent technical replicates from which mean inhibition zone values were computed. Interpretation of inhibition zones was based on CLSI guidelines (CLSI, 2020) [41], using criteria established for *Staphylococcus* spp., *Enterococcus* spp., and *Enterobacterales*. Due to the absence of CLSI breakpoints for Listeria, the criteria applied in this study represent phenotypic inhibition zone categories rather than clinical susceptibility interpretations. Quality control strains employed in the study included *Escherichia coli* ATCC 25922, *Staphylococcus aureus* ATCC 29213, and *L. innocua* NCTC 11288. Microsoft Excel software was used to analyze the data. The results were expressed as proportions with 95% confidence intervals. A significance level of 0.05 was used.

### 3.3. Detection of Antibiotic Resistance—Associated Genes

Isolates exhibiting resistance or intermediate resistance in phenotypic analyses were selected for the detection of antimicrobial resistance genes. The presence of resistance genes was assessed by polymerase chain reaction (PCR). Each PCR mixture (10 µL final volume) contained 0.2 U of RUN polymerase (A&A Biotechnology, Gdansk, Poland) with 1× concentrated reaction buffer, 0.2 mM dNTPs (A&A Biotechnology, Gdansk, Poland), and sequence-specific primers designed based on published nucleotide sequences (details provided in Supplementary Table S1), synthesized by Genomed S.A. (Warsaw, Poland). Genomic DNA was added at a concentration of 10 ng per reaction. PCR products were separated by electrophoresis on 2–2.5% agarose gels containing Midori Green Advance (NIPPON Genetics EUROPE, Düren, Germany), at a final concentration of 1×. One representative product from each primer pair, corresponding to the expected amplicon size, was excised from an agarose gel following electrophoresis and purified using the Gel-Out Concentrator kit (A&A Biotechnology, Gdańsk, Poland). The purified products were then subjected to automated Sanger sequencing based on capillary electrophoresis (Applied Biosystems chemistry) at Genomed S.A. (Warsaw, Poland). Sequencing results were inspected manually for quality, and consensus sequences were compared with reference gene sequences deposited in the NCBI database using the BLASTN 2.15.0 tool.

## 4. Results

### 4.1. Antibiotic Susceptibility

Antibiotic susceptibility tests for 51 isolates of *L. innocua* were conducted using the disk diffusion method. Among the 18 antibiotics selected for this study, those commonly used in the treatment of listeriosis were included. Table 1 summarizes the antimicrobial susceptibility profiles of isolated *L. innocua*.

In this study, the antibiotic susceptibility of 51 *L. innocua* isolates was assessed against 18 antimicrobial agents, revealing heterogeneous resistance patterns. Complete susceptibility (100%) was observed to ampicillin, benzylpenicillin, chloramphenicol, gentamicin, rifampin, trimethoprim-sulfamethoxazole, and vancomycin. High susceptibility was also recorded for ciprofloxacin, erythromycin, meropenem, trimethoprim, with only sporadic intermediate isolates (2–6%). Moderate susceptibility levels were found for streptomycin (88% susceptible) and tetracycline (84% susceptible), with resistance detected in 10% of isolates for each. The lowest activities were observed for nitrofurantoin, clindamycin and linezolid, with only 4% of isolates fully susceptible, while the majority exhibited intermediate or resistant phenotypes. Universal resistance (100%) was noted for oxacillin and cefotaxime.

The isolates showed 17 different antibiotic resistance patterns (Table 2). The most observed pattern was intermediate resistance to clindamycin, linezolid and nitrofurantoin as well as resistance to cefotaxime and oxacillin (n = 15, 29%). Analysis of multidrug resistance (MDR), defined as nonsusceptibility to at least one antimicrobial agent in three or more antibiotic classes [27], demonstrated among the isolates revealed that all 51 isolates (100%) were resistant to two different antibiotics, and 25 isolates (49%) were resistant to three different antibiotics. Furthermore, six isolates (12%) exhibited resistance to four antibiotics, while five isolates (10%) were resistant to five antibiotics. Additionally, two isolates (4%) were resistant to six and seven antibiotics, respectively.

### 4.2. Presence of Antibiotic Resistance—Associated Genes

In the case of tested antibiotic resistance—associated genes, all the *L. innocua* isolates included in the study were positive in PCR with amplicons of expected length, in accordance with the literature references. The results were further confirmed with sequencing and BLAST 2.15.0 analyses. The summarized detection results are presented in Table 3.

## 5. Discussion

In this study, the disk diffusion method was used, as it remains the most widely applied approach for preliminary screening of antimicrobial susceptibility and enables direct comparison with previously published *L. innocua* resistance data. Determination of minimum inhibitory concentrations (MIC) is another commonly used method that offers quantitative information complementary to disk diffusion. Although MIC testing was not included in the present work, integrating MIC determinations in subsequent analyses would further strengthen the evaluation of antimicrobial susceptibility in *L. innocua* and provide a more detailed and comprehensive characterization of resistance phenotypes. Future investigations will therefore incorporate MIC measurements to complement the disk diffusion results.

To date, several studies have investigated the antimicrobial susceptibility of *L. innocua* isolates obtained from various food sources and geographic regions. However, direct comparison of results remains challenging due to methodological heterogeneity across publications. Differences in interpretive criteria, whether EUCAST or CLSI, and the choice of microbiological media result in variability that can influence zone diameter measurements and, consequently, susceptibility classification. Although EUCAST provides interpretive criteria for *L. monocytogenes*, these are limited to only four antibiotics, rendering them insufficient for comprehensive susceptibility profiling. To ensure methodological consistency across all antibiotics tested, interpretive standards established by the CLSI were employed. This approach has been widely adopted in previous studies involving both *L. monocytogenes* and *L. innocua*, particularly when species-specific breakpoints were unavailable or incomplete [9,12,21,24,26,27,33,43,44,45]. Specifically, CLSI breakpoints developed for *Enterococcus* spp., *Staphylococcus* spp., and *Enterobacterales* were used as reference points for zone diameter interpretation. A similar approach has been reported in other publications, where authors either state the reference organisms used, most commonly *Staphylococcus* spp. and *Enterococcus* spp. [9,15,27,33], or omit such details, leaving the exact interpretive criteria unspecified. On the one hand, the use of these reference organisms facilitates comparison with previously published studies and maintains methodological consistency across investigations. On the other hand, applying interpretive criteria developed for bacteria other than *Listeria* provides only an approximate indication of the antimicrobial susceptibility profile of *L. innocua* and may not fully reflect its true phenotypic behavior. Nevertheless, this strategy remains one of the most practical and commonly applied approaches for interpreting susceptibility data in *Listeria* species in the absence of species-specific breakpoints. In contrast, only a limited number of studies rely exclusively on EUCAST guidelines [11].

Although *L. innocua* is not the primary target organism in either CLSI or EUCAST guidelines, the use of established breakpoints for phylogenetically related bacterial groups is a widely accepted alternative in the absence of species-specific standards. Nevertheless, this remains a limitation of the present study. In addition to interpretive criteria, the choice of microbiological media used for susceptibility testing varies across studies. Although EUCAST methodology recommends a modified medium, Mueller–Hinton agar supplemented with 5% defibrinated horse blood and 20 mg/L β-NAD, for fastidious organisms, as well as incubation in a 5% CO_2_ atmosphere. Some authors have followed EUCAST recommendations and used the modified Mueller–Hinton medium [15,16,26], while others have employed standard Mueller–Hinton agar under ambient air conditions, as was performed in the present study [11,12,27]. Methodological differences, encompassing both interpretive criteria and culture conditions, can influence susceptibility results and should be considered when comparing data across studies. Inconsistent application of breakpoints and culture conditions may result in over- or underestimation of resistance rates, thereby complicating the detection of true epidemiological patterns. This is particularly relevant for *L. innocua*, for which species-specific interpretive criteria are lacking. To our knowledge, the present study provides the first detailed characterization of antimicrobial resistance profiles in *L. innocua* isolates from meat products and meat-processing environments in Poland. *L. innocua* isolates displayed heterogeneous antimicrobial resistance patterns, with generally high susceptibility to several clinically important antibiotics, but also the occurrence of isolates showing broad resistance profiles.

A total of 18 antibiotics representing 11 major classes of antimicrobial agents were analyzed. The selection aimed to cover the most relevant antimicrobial classes used in human and veterinary medicine, allowing for the evaluation of potential cross-resistance and facilitating comparison with previously published studies on *L. innocua* [14,15,16].

In this study, complete susceptibility (100%) was observed for seven of the 18 antibiotics tested: ampicillin, benzylpenicillin, chloramphenicol, gentamicin, rifampin, trimethoprim-sulfamethoxazole, and vancomycin. This finding highlights the sustained activity of several clinically important drugs against *L. innocua*. Ampicillin and benzylpenicillin remain the cornerstone of listeriosis treatment. Their full efficacy in our isolates is in agreement with reports from the United States, Spain, and Italy, where susceptibility typically exceeds 95% [9,11,12]. However, lower susceptibility rates have been described in Malaysia [27] and South Africa [45], suggesting possible regional variation linked to antibiotic usage patterns. Vancomycin, a glycopeptide considered an important last-resort option against Gram-positive infections, was uniformly effective. Similar results have been reported in Europe and North America [9,11], though reduced susceptibility has occasionally been described in Asia [16,27]. The absence of glycopeptide resistance in our isolates suggests that *L. innocua* does not currently represent a clinical concern in this regard. Chloramphenicol also showed full efficacy, consistent with studies from the United States and Turkey [9,15]. Although its clinical use is limited due to toxicity, its activity supports its value as a research marker of *Listeria* susceptibility. Gentamicin exhibited 100% susceptibility, supporting its role as an effective adjunct in combination therapy for listeriosis. Comparable results have been reported across several regions [15,45], although occasional reduced activity has been noted in Turkey [46]. Rifampin also retained complete activity, consistent with findings from Iran and Malaysia [16,27]. Although not a standard treatment option for listeriosis, its efficacy indicates preserved sensitivity among environmental and food-derived isolates. Finally, trimethoprim-sulfamethoxazole (SXT) showed full susceptibility. This is consistent with most published studies [16,33], although resistance has occasionally been documented in small isolate collections from Poland [24]. SXT is considered the main alternative therapy for patients allergic to β-lactams, and its retained efficacy remains clinically reassuring.

Several agents displayed high overall activity, with only sporadic reduced susceptibility: ciprofloxacin (96%), erythromycin (98%), meropenem (94%), and trimethoprim (96%). Fluoroquinolones such as ciprofloxacin showed nearly universal susceptibility, although isolated resistant strains were detected. Similar high activity has been reported in the United States [33] and South Africa [44], but much lower rates in Turkey [15] and Iran [26]. In the current study, it was demonstrated that erythromycin was highly effective (98% susceptible), consistent with reports from Turkey and the United States [9,45]. However, markedly lower susceptibility has been noted in China (64%; [21]) and in Poland (80%; [24]). Resistance in *Listeria* is typically mediated by *erm* genes encoding 23S rRNA methyltransferases, though resistant *L. innocua* strains sometimes lack these determinants, suggesting alternative mechanisms. Meropenem also demonstrated near-complete efficacy, with only one isolate showing reduced susceptibility. High activity has been described in Spain [11] and Turkey [15], but a Polish study of milk-derived isolates reported resistance in up to 40% of strains [24]. Trimethoprim, when tested alone, was active against 98% of isolates, although two isolates showed reduced susceptibility. Trimethoprim remains a highly effective antibiotic against *Listeria*, as demonstrated in the literature [15], showing complete susceptibility.

Two antibiotics exhibited only moderate efficacy: streptomycin (88% susceptible) and tetracycline (84% susceptible). Streptomycin resistance was observed in 10% of isolates, with an additional 2% showing reduced susceptibility. Comparable levels have been reported in Iran [16], though much higher resistance was found in China (24%; [21]) and South Africa (51%; [45]). In *Listeria*, resistance is often linked to *strA* and *strB* genes, although their presence in *L. innocua* has not been confirmed. Tetracycline susceptibility was similarly reduced, with 10% resistant isolates and 6% showing intermediate responses. International data show wide variability, from full susceptibility in Turkey [15] to high resistance in Iran (up to 86%; [16]). The primary resistance determinant is *tet*(*M*), commonly located on conjugative transposons of the Tn916/Tn1545 family [34]. Its high prevalence reflects the extensive use of tetracyclines in veterinary medicine and animal feed, positioning *L. innocua* as an important reservoir of *tet*(*M*) within the *Listeria* genus [23,47]. For the antibiotic nitrofurantoin, 25% of isolates remained susceptible, while 67% showed reduced susceptibility and 8% were resistant. Higher resistance levels have been reported in Turkey (35%; [15]), suggesting regional variability.

The lowest activity was observed for clindamycin and linezolid. For clindamycin, 78% of isolates exhibited reduced susceptibility and 22% were resistant. Similar high resistance has been observed worldwide, reaching 95% in the United States [9], 93% in China [27], and 82% in Turkey [15]. Resistance is usually associated with *lnuA* and *lnuB*. The current study confirmed that one isolate was found to carry the *lnuA* gene, while none of the isolates possessed the *lnuB* gene. There is a lack of reliable literature sources confirming the presence of *lnuA* and *lnuB* genes in *L. innocua*, suggesting alternative mechanisms such as enzyme inactivation. Linezolid also demonstrated poor activity: 73% of isolates were intermediately susceptible, while 24% were resistant. These results contrast sharply with findings from Spain [11] and Turkey [15], where susceptibility often approached 100%. In *L. monocytogenes*, resistance has been linked to *cfr*, *optrA*, and *poxtA* genes [44], but these determinants have not yet been reported in *L. innocua*. Similar results were obtained in this study, as none of the isolates carried out the mentioned genes.

Finally, the findings of this study indicate that all isolates were resistant to oxacillin and cefotaxime. This observation is consistent with reports from Spain and Turkey [11,15], though some variability exists, with higher susceptibility observed in Italy (37%; [12]) and complete susceptibility in one Turkish study [46]. The literature has indicated that resistance to oxacillin may be associated with the presence of the *mecA* gene. The current study confirmed that, two isolates carried this gene, while the remaining ones did not; therefore, the exact mechanism of resistance to this antibiotic cannot be determined.

The antimicrobial susceptibility profiles of *L. innocua* isolates obtained in this study generally align with findings from other European countries. Uniform susceptibility to clinically important antibiotics like ampicillin, gentamicin, erythromycin, trimethoprim/sulfamethoxazole, and vancomycin, was observed both in this study and in reports from Spain and Italy, indicating a stable sensitivity pattern across these regions. However, comparable data from countries geographically closer to Poland are not available, this gap in the literature highlights the uniqueness and importance of the current study, as it provides essential baseline information on antimicrobial resistance in *L. innocua* in this part of Europe. On the contrary, other global studies from Asia, Africa, and North America demonstrated greater variability in resistance profiles, particularly tetracycline. These differences may be attributed to regional antibiotic usage practices, environmental factors, and strain differences. Importantly, the antibiotics commonly used in the treatment of listeriosis showed high phenotypic susceptibility among the *L. innocua* isolates, indicating that these agents remain effective against this species based on the obtained laboratory results. However, the fact that bacteria can develop resistance mechanisms or acquire resistance through horizontal gene transfer from other bacterial species has contributed to the global rise in antimicrobial resistance and may significantly complicate efforts to control bacterial infections. In addition to the presence of specific resistance genes, other mechanisms may also contribute to antimicrobial resistance, such as alterations in cell membrane permeability, modification of antibiotic target sites, or enzymatic degradation of the antimicrobial agent. Therefore, to fully elucidate the mechanisms underlying resistance, it is essential to investigate not only genetic determinants, but also potential physiological and biochemical processes involved in resistance expression [1]. The presence of multidrug-resistant strains and regional differences in resistance patterns highlights the need for ongoing monitoring of antimicrobial usage. In this study, resistance genes associated with clindamycin (*lnuA*, *lnuB*), linezolid (*cfr*, *optrA*, *poxtA*), and oxacillin (*mecA*) were analyzed, as these antibiotics showed the most variable and interesting phenotypic results. Cefotaxime was not included because *Listeria* spp. are known to exhibit natural resistance to this antibiotic. Although only a limited number of resistance genes were examined, this represents a limitation of the present study. Future investigations should include a broader range of resistance genes, covering additional antibiotic classes and alternative variants of the genes already tested, to provide a more comprehensive understanding of resistance mechanisms in *L. innocua*.

## 6. Conclusions

The findings of this study provide valuable insights into the antimicrobial resistance patterns of *L. innocua*, a species often overlooked in resistance surveillance compared to *L. monocytogenes*. Consistent with previous reports, our results indicate that resistance profiles in *L. innocua* are influenced by the type of food product and by geographical factors reported globally. However, due to the lack of detailed regional data, this aspect could not be specifically evaluated in the present study. This underscores the importance of localized monitoring, as resistance patterns vary considerably between isolates from different sources. The results obtained expand the knowledge of the pathogen’s prevalence in the study region and highlight a significant public health concern that poses a threat to consumer health. Although data on *L. innocua* from this region are currently limited, this emphasizes the importance of the present study in providing valuable baseline information and helping to fill a critical knowledge gap. Each set of isolates contributes uniquely to the global understanding of antimicrobial resistance. Although *L. innocua* has traditionally been considered non-pathogenic, it has demonstrated the capacity to harbor clinically relevant resistance genes and may serve as a reservoir for horizontal gene transfer to other bacteria, including pathogenic *Listeria* species. The coexistence of *L. innocua* with other *Listeria* spp. in the same food sample or processing environment could further facilitate the dissemination of antimicrobial resistance between these species. The potential for resistance acquisition—even in environments where resistance is not yet apparent—emphasizes the need for proactive surveillance. Future research should focus on whole-genome sequencing (WGS) to identify novel resistance genes and assess the potential for horizontal gene transfer. While WGS offers a comprehensive view of genetic mechanisms, PCR-based screening remains a rapid, affordable, and accessible tool for detecting known resistance genes. Together, these complementary approaches can enhance the understanding of resistance dynamics in *Listeria* species. Therefore, antimicrobial resistance monitoring should not be limited to *L. monocytogenes* but should also include *L. innocua* as part of a comprehensive strategy to combat antibiotic resistance. Incorporating *L. innocua* into routine resistance surveillance programs may function as an early warning system and a critical component in preserving the efficacy of antimicrobial agents in the context of food safety and public health.

## Figures and Tables

**Table 1:** Results of antibiotic susceptibility tests for *L. innocua* isolates conducted using the disk diffusion method.

Antibiotic Class	Antibiotic	Interpretation Criteria	Sources of Isolates		Total
			Food (n = 32)	Food Processing Environments (n = 19)	
β-lactams	AMP	S (≥17 mm)	32 (100%)	19 (100%)	**51 (100%)**
		I (N/A ^1^)	0	0	**0**
		R (≤16 mm)	0	0	**0**
	P	S (≥15 mm)	32 (100%)	19 (100%)	**51 (100%)**
		I (N/A ^1^)	0	0	**0**
		R (≤14 mm)	0	0	**0**
	CE	S (≥26 mm)	0	0	**0**
		I (23–25 mm)	0	0	**0**
		R (≤22 mm)	32 (100%)	19 (100%)	**51 (100%)**
	MEM	S (≥23 mm)	30 (93%)	18 (95%)	**48 (96%)**
		I (20–22 mm)	2 (7%)	2 (5%)	**3 (4%)**
		R (≤19 mm)	0	0	**0**
	OX	S (≥18 mm)	0	0	**0**
		I (N/A ^1^)	0	0	**0**
		R (≤17 mm)	32 (100%)	19 (100%)	**51 (100%)**
Phenicols	C	S (≥18 mm)	32 (100%)	19 (100%)	**51 (100%)**
		I (13–17 mm)	0	0	**0**
		R (≤12 mm)	0	0	**0**
Fluoroquinolones	CIP	S (≥21 mm)	32 (100%)	17 (90%)	**49 (96%)**
		I (16–20 mm)	0	2 (10%)	**2 (4%)**
		R (≤15 mm)	0	0	**0**
Lincosamides	DA	S (≥21 mm)	0	0	**0**
		I (15–20 mm)	23 (72%)	17 (90%)	**40 (78%)**
		R (≤14 mm)	9 (22%)	2 (10%)	**11 (22%)**
Macrolides	E	S (≥23 mm)	32 (100%)	18 (95%)	**50 (98%)**
		I (15–20 mm)	0	0	**0**
		R (≤14 mm)	0	1 (5%)	**1 (2%)**
Aminoglycosides	CN	S (≥15 mm)	32 (100%)	19 (100%)	**51 (100%)**
		I (13–14 mm)	0	0	**0**
		R (≤12 mm)	0	0	**0**
	S	S (≥15 mm)	24 (79%)	19 (100%)	**45 (88%)**
		I (12–14 mm)	1 (3%)	0	**1 (2%)**
		R (≤11 mm)	5 (18%)	0	**5 (10%)**
Oxazolidinones	LNZ	S (≥26 mm)	1 (3%)	1 (5%)	**2 (4%)**
		I (23–25 mm)	24 (75%)	14 (74%)	**37 (73%)**
		R (≤22 mm)	7 (22%)	5 (26%)	**12 (24%)**
Nitrofurans	F	S (≥17 mm)	5 (16%)	8 (42%)	**13 (25%)**
		I (15–16 mm)	23 (72%)	10 (53%)	**34 (67%)**
		R (≤14 mm)	4 (13%)	1 (5%)	**4 (8%)**
Rifamycins	RA	S (≥20 mm)	32 (100%)	19 (100%)	**51 (100%)**
		I (17–19 mm)	10	0	**0**
		R (≤16 mm)	0	0	**0**
Tetracyclines	TE	S (≥19 mm)	26 (72%)	19 (100%)	**45 (88%)**
		I (15–18 mm)	0	0	**0**
		R (≤14 mm)	6 (28%)	0	**6 (12%)**
Folate inhibitors	TMP	S (≥16 mm)	31 (98%)	19 (100%)	**49 (96%)**
		I (11–15 mm)	1 (2%)	0	**2 (4%)**
		R (≤10 mm)	0	0	**0**
	SXT	S (≥16 mm)	32 (100%)	19 (100%)	**51 (100%)**
		I (11–15 mm)	0	0	**0**
		R (≤10 mm)	0	0	**0**
Glycopeptides	VA	S (≥17 mm)	32 (100%)	19 (100%)	**51 (100%)**
		I (15–16 mm)	0	0	**0**
		R (≤14 mm)	0	0	**0**

N/A ^1^—Not apply, S—Susceptible, I—Intermediate, R—Resistance; AMP—Ampicillin, P—Benzylpenicillin, CE—Cefotaxime, C—Chloramphenicol, CIP—Ciprofloxacin, DA—Clindamycin, E—Erythromycin, CN—Gentamicin, LNZ—Linezolid, MEM—Meropenem, F—Nitrofurantoin, OX—Oxacillin, RA—Rifampin, S—Streptomycin, TE—Tetracycline, TMP—Trimethoprim, SXT—Trimethoprim/sulfamethoxazole, VA—Vancomycin.

**Table 2:** Antimicrobial resistance profiles of *L. innocua* isolates.

Antibiotic Resistance	Sources of Isolates		Total Number (%) of Resistant Isolates	Profile
	Food (n = 32)	Food Processing Environments (n = 19)		
DA (I), LNZ (I), F (I), CE (R), OX (R)	7 (22%)	8 (42%)	15 (29%)	**A**
DA (I), F (I), LNZ (R), CE (R), OX (R)	4 (13%)	4 (21%)	8 (16%)	**B**
DA (I), LNZ (I), CE (R), OX (R)	4 (13%)	2 (11%)	6 (12%)	**C**
LNZ (I), F (I), DA (R), CE (R), OX (R)	4 (13%)	-	4 (8%)	**D**
DA (I), LNZ (R), CE (R), OX (R)	3 (9%)	-	3 (6%)	**E**
LNZ (I), DA (R), CE (R), OX (R)	1 (3%)	1 (5%)	2 (4%)	**F**
DA (I), LNZ (I), MEM (I), F (I), CE (R), OX (R)	1 (3%)	1 (5%)	2 (4%)	**G**
LNZ (I), DA (R), F (R), TE (R), S (R), CE (R), OX (R)	2 (6%)	-	2 (4%)	**H**
DA (I), F (I), CE (R), OX (R)	-	1 (5%)	1 (2%)	**I**
DA (I), F (I), TE (R), CE (R), OX (R)	1 (3%)	-	1 (2%)	**J**
DA (I), LNZ (I), CIP (I), CE (R), OX (R)	1 (3%)	-	1 (2%)	**K**
DA (I), LNZ (I), F (I), CIP (I), CE (R), OX (R)	-	1 (5%)	1 (2%)	**L**
DA (I), LNZ (I), F (R), E (R), CE (R), OX (R)	-	1 (5%)	1 (2%)	**M**
DA (I), CIP (I), F (I), LNZ (R), CE (R), OX (R)	-	1 (5%)	1 (2%)	**N**
LNZ (I), F (I), DA (R), TE (R), S (R), CE (R), OX (R)	1 (3%)	-	1 (2%)	**O**
LNZ (I), DA (R), F (R), TE (R), S (R), TMP (R), CE (R), OX (R)	1 (3%)	-	1 (2%)	**P**
LNZ (I), MEM (I), DA (R), F (R), TE (R), S (R), TMP (R), CE (R), OX (R)	1 (3%)	-	1 (2%)	**R**

I—Intermediate, R—Resistance; CE—Cefotaxime, CIP—Ciprofloxacin, DA—Clindamycin, E—Erythromycin, LNZ—Linezolid, MEM—Meropenem, F—Nitrofurantoin, OX—Oxacillin, S—Streptomycin, TE—Tetracycline, TMP—Trimethoprim. Profile: A–R denote resistance profiles of *L. innocua* isolates according to their phenotypic susceptibility patterns.

**Table 3:** Detection of resistance genes results.

Antibiotic	Antibiotic Resistance Genes	Number of Isolates (%)		Total Positive
		Food n = 32 (62.7%)	Food Processing Environments n = 19 (37.3%)	
DA	*lnuA*	0	1 (5%)	**1 (2%)**
	*lnuB*	0	0	**0**
LNZ	*cfr*	0	0	**0**
	*optrA*	0	0	**0**
	*poxtA*	0	0	**0**
OX	*mecA*	0	2 (11%)	**2 (4%)**

DA—Clindamycin, LNZ—Linezolid, OX—Oxacillin.

## Data Availability

The original contributions presented in the study are included in the article (and Supplementary Material); further inquiries can be directed to the corresponding authors.

## Supplementary Materials

The following supporting information can be downloaded at https://www.mdpi.com/article/10.3390/genes16121455/s1. Table S1. Sequences of primers used for detection of antibiotic resistance genes.
