# Supplementary material for: Antibiotic Resistance Profiles and Genetic Determinants of Listeria innocua Isolated from Food Sources in Poland
Source: Genes (Basel). 2025 Dec 5;16(12):1455. doi: 10.3390/genes16121455 (PMC12732994; doi:10.3390/genes16121455)
Supplement: Supplementary file 1 [file genes-16-01455-s001.zip › genes-3986021-supplementary.pdf]

Table S1. Sequences of primers used for detection of antibiotic resistance genes.

Antibiotic	Gene names	Primers' Sequence	Primer Concentration [μM]	Amplicon Length [bp]	Cycling Conditions	References
DA	*lnuA*	GGTGGCTGGGGGGTAGATGTATTAACTGG<br>GCTTCTTTTGAAATACATGGTATTTTTCGATC	0.4	323	94°C 5 min; (94°C 60 s; 59°C 60 s; 72°C 2 min) × 35; 72°C 5 min	[43]
	lnuB	CCTACCTATTGTTTGTGGAA<br>ATAACGTTACTCTCCTATTC	1.0	405	94°C 5 min; (94°C 60 s; 54°C 60 s; 72°C 2 min) × 35; 72°C 5 min	
LNZ	*cfr*	TGAAGTATAAAGCAGGTTGGGAGTC<br>AACCATATAATTGACCACAAGCAGC	0.1	746	96°C 2 min; (96°C 30 s; 50°C 30 s; 72°C 30 s) × 30; 72°C 5 min	[44]
	optrA	TACTTGATGAACCTACTAACCA<br>CCTTGAACTACTGATTCTCGG	0.1	422		
	poxtA	AAAGCTACCCATAAAATATC<br>TCATCAAGCTGTTCGAGTTC	0.1	533		
OX	*mecA*	GTGGAAAAGGCGAAGATGGC<br>TCAAGTCCTGTTGCTCGTGA	0.2	199	94°C 5 min; (98°C 10 s; 59°C 30 s; 72°C 60 s) × 30; 72°C 7 min	[8]

DA-Clindamycin, LNZ-Linezolid, OX-Oxacillin
